# Commentary: Case report: Associated ocular adverse reactions with inactivated COVID-19 vaccine in China

**DOI:** 10.3389/fmed.2022.991862

**Published:** 2022-09-21

**Authors:** Yusuke Kameda, Yutaka Kaneko, Megumi Sugai, Karin Ishinabe, Nichika Fukuoka

**Affiliations:** ^1^Yotsuya-sanchome Ekimae Eye Clinic, Tokyo, Japan; ^2^Department of Ophthalmology and Visual Sciences, Faculty of Medicine, Yamagata University, Yamagata, Japan

**Keywords:** COVID-19 vaccination, herpes keratitis, herpes simplex virus (HSV), varicella zoster virus (VZV), PCR, COVID-19

## Introduction

We read the article by Pang et al. with considerable interest, a case series study that provided interesting novel insights into ocular adverse events that occurred after administering the coronavirus disease 2019 (COVID-19) vaccine (1). Although we congratulate the authors for a valuable case presentation, we had some doubts regarding the four cases of keratitis. Therefore, through many years of our experience regarding herpetic eye disease, we would like to provide critical comments for each case.

## Case 3

There were no images or descriptions of the findings in the case presentation. Even if the article type explained the selected case, the authors should add the characteristic appearances and outcomes in the table, such as the study by Bolletta et al. (2). While insufficient disclosure of information about case 3 made it difficult for the readers to imagine the causative factors of keratitis, we assumed that the underlying pathogenesis in this case was herpes virus because the patient was treated with ganciclovir ophthalmic gel. The diagnosis of recurrent or typical herpes keratitis can be made based only on clinical findings; however, no history of this disease and evidence suggesting a typical case were noted in case 3 (3, 4). Therefore, the polymerase chain reaction test is ideal for accurate diagnosis of atypical or complicated cases (3, 5, 6).

## Case 4

The authors observed a dendritic ulcer with terminal bulbs in the central cornea on slit-lamp examination of Supplementary Figure 1A in Pang et al.'s article. However, we could not confirm this finding due to low-resolution images. Moreover, about 3 months before the first examination, the patient had received COVID-19 vaccination; a relatively long interval between the first presentation and vaccination indicated that the likelihood of other causative factors was higher. Thus, it was very difficult to determine whether the development of keratitis in case 4 was affected by COVID-19 vaccination.

## Case 5

The authors diagnosed case 5 as keratitis, although they observed a red eye in the patient. This finding suggested that keratoconjunctivitis, as in case 2, was preferable to the aforementioned diagnosis. As shown in Figure 2 of Pang et al.'s article, the cutaneous reaction seemed to be a typical varicella zoster virus reactivation. Although reactivation following COVID-19 vaccination was well-documented even at the study period, the authors never addressed the relationship; therefore, this should be discussed with more references (7–9). Moreover, similar to case 4, we felt that the images in Supplementary Figure 2 of Pang et al.'s article showed the slit-lamp examination of case 5 was too low in resolution and long between the onset and the photographing date to draw a positive conclusion.

## Case 7

Like case 3, despite no images or description of findings, the authors speculated that the causative factor of keratitis was herpes virus due to the administration of ganciclovir ophthalmic gel. The onset time was 14 days after vaccination, which could be affected by other influences because of a longer duration than herpes keratitis cases following COVID-19 vaccination, as documented in previous reports (2, 4, 5, 10). Incidentally, at our institution, widespread COVID-19 vaccinations afforded some opportunities to examine the occurrence of herpes keratitis in patients who had recently received the vaccination; the onset time in all the patients was within a few days of vaccination.

## Conclusion

**Figure 1:**
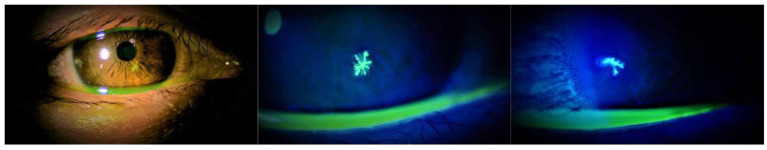
Slit-lamp examination of the right eye of the patient showing a herpes keratitis that occurred on the day after the third dose of BNT162b2 (Pfizer). Photos were taken 2 days after the vaccination.

In the COVID-19 pandemic era, accumulated case series and isolated case reports have suggested that herpes keratitis could occur after COVID-19 vaccination (2, 4–6, 10). We also presented images of herpes keratitis that occurred on the day after COVID-19 vaccination (Figure 1). The picture quality seemed sufficient to explain the two dendritic ulcers with terminal bulbs. In contrast, it is difficult to determine whether the relationship between herpes keratitis that occurred immediately after inoculation and the vaccine was causative or coincidental because this disease is a common illness. Moreover, even if case series and isolated case reports with detailed descriptions of findings and images that can convince readers are accumulated, these study types are generally regarded as “low level” evidence (11). Therefore, future study with high evidence level will be required to determine whether the COVID-19 vaccination was associated with the incidence of herpes keratitis.

## Author contributions

YKam researched the study by Pang et al., wrote the manuscript, and contributed to the critical comments. YKan, MS, KI, and NF helped in the reviewing and editing of the manuscript. As the corresponding author and guarantor of this manuscript, YKam takes full responsibility for the work as a whole. All authors have read and agree to the published version of the manuscript and approved the final version of the manuscript.

## Conflict of interest

The authors declare that the research was conducted in the absence of any commercial or financial relationships that could be construed as a potential conflict of interest.

## Publisher's note

All claims expressed in this article are solely those of the authors and do not necessarily represent those of their affiliated organizations, or those of the publisher, the editors and the reviewers. Any product that may be evaluated in this article, or claim that may be made by its manufacturer, is not guaranteed or endorsed by the publisher.
